# Editorial: Women in science: Public mental health 2022

**DOI:** 10.3389/fpubh.2022.1123506

**Published:** 2023-01-05

**Authors:** Yuka Kotozaki, Elisa Harumi Kozasa

**Affiliations:** ^1^Iwate Medical University, Yahaba, Japan; ^2^Hospital Israelita Albert Einstein, São Paulo, Brazil

**Keywords:** public health, public mental health, mental health, women's health, women's mental health, global mental health, inequality, gender

This Research Topic forms volume II in the series “Women in Science: Public Mental Health”. Although women now outnumber men among college graduates, men continue to outnumber women in most science, technology, engineering, and mathematics (STEM) fields and majors (1, 2). Additionally, there is a lack of representation of women in senior positions in public health (3). According to 2016 data from the UNESCO Institute for Statistics, < 30% of researchers in STEM are women (4). In the field of public mental health, there are many highly influential and successful women who are contributing to the field and tackling important questions. This Research Topic focuses on highlighting women's contributions to public health, specifically in the field of mental health.

Five of the papers submitted to the Research Topic by an international set of researchers were deemed suitable for publication after a thorough peer review process. The following is a summary of the major results of each of these manuscripts.

In a brief research report that constitutes the first article in this special Research Topic, Björkstedt et al. examine the impact of preconception severe mental disorders on pregnancy outcomes in primiparous women. Of all women participating in this study, 3.4% had at least one psychiatric diagnosis. The most common psychiatric diagnoses were depression and anxiety disorders, and the most common comorbidity was the combination of depression and anxiety disorders. However, the wellbeing of these women's newborns was good. This result suggests that newborns of primiparous women with severe mental diseases seem to be born in good health. Additionally, primiparous women with severe mental health disorders are often members of groups with lower socioeconomic status. The preconception prevalence of severe mental diseases is low, and comorbidity is common.

In the next article, Lu et al. describe the prevalence and correlates of psychotic-like experiences (PLEs) among pregnant women without previous psychiatric history in each trimester. In their sample, 37.2% of pregnant women experienced at least one episode of PLEs, while 4.3% reported “often” having PLEs. More pregnant women experienced PLEs, delusional experiences, and hallucinatory experiences in the first two trimesters than in the third trimester. Factors associated with a higher risk of more frequent PLEs include a rural setting, unplanned pregnancy, parity 1, and higher Edinburgh postnatal depression scale (EPDS) score. Strong positive correlations with frequency of PLEs were observed for scores on the seven-item generalized anxiety disorder scale and the EPDS. The authors suggest that episodes of PLEs are common among Chinese pregnant women; however, only a small proportion experience persistent PLEs. It is vital to pay attention to women with a risk of psychosis in pregnancy.

In the third article presented in this Research Topic, Christopher et al. examine the results of list experiment and direct questions about experiences of physical and sexual intimate partner violence (IPV) from a 2017 cross-sectional survey among 2,299 adults aged 40 years or older in Dar es Salaam. Their findings indicate that the list experiment estimated a higher prevalence of IPV in all cases except for that of physical violence experienced by women. The authors suggest that this study contributes to the estimation of the prevalence of IPV. If the list experiment questions yield an unbiased estimate, the findings also suggest that women openly report experiencing physical IPV, while IPV experienced by men is underreported and understudied.

In the next article, Mohammadabadi et al. examine the factors affecting adherence to pharmacological and psychotherapeutic treatment and patients' attitudes toward medication, and assess medication and treatment adherence in patients. Evidence collected using the Drug Attitude Inventory-10 showed that 54 participants had negative attitudes toward medication, while 38 participants exhibited negative attitudes toward psychotherapy treatment. Additionally, the percentage of patients who showed good treatment adherence was higher for psychotherapy than for medication. The most common reasons for discontinuation of treatment were medication side effects, dissatisfaction with the therapist, and fear of medication dependence. Patients with higher levels of education and a positive history of hospitalization on a psychiatric ward exhibited better adherence to psychotherapy. The authors suggest that attitudes toward psychotherapy are more favorable than attitudes toward pharmacotherapy among patients with borderline personality disorder.

Finally, Pei et al. examine the factors influencing prenatal mental disorders and provide a scientific basis for guidance on pregnant women's mental health. Participants in different symptom classes reported differently on their experiences of the perinatal period, marriage satisfaction, their relationships with in-laws, their relationships with friends, underlying diseases, and use of birth control pills. Three latent classes were identified: a high-symptoms group, a moderate-symptoms group, and a low-symptoms group. Pregnant women in the third trimester, with poor relationships with their in-laws and friends, and who had basic diseases tended to be classified in the high-symptoms group rather than the low-symptoms group. Also, those who have a lack of friend relationship, and take birth control pills. The authors suggest that in order to ensure the smooth progress of pregnancy and promote the physical and mental health of pregnant women, psychological screening and psychological interventions should be strengthened.

In conclusion, the editors wish to thank all the authors, the reviewers, and the editorial board members for contributing to this Research Topic. We hope this Research Topic might inspire future and novel research approaches in the field of mental health.

## Author contributions

Both authors listed have made a substantial, direct, and intellectual contribution to the work and approved it for publication.

## References

[1] National Research Council. A Framework for K-12 Science Education: Practices, Crosscutting Concepts, and Core Ideas. Washington, DC: The National Academies Press (2012).

[2] Dasgupta N Stout JG. Girls and women in science, technology, engineering, and mathematics: STEMing the tide and broadening participation in STEM careers. Policy Insights Behav Brain Sci. (2014) 1:21–9. 10.1177/2372732214549471

[3] Pérez-Sánchez S Madueño SE Montaner J. Gender gap in the leadership of health institutions: the influence of hospital-level factors. Health Equity. (2021) 5:521–5. 10.1089/heq.2021.0013 34476325 PMC8409238

[4] Lewis J Schneegans S Straza T. UNESCO Science Report: The Race Against Time for Smarter Development. Vol. 2021. Paris: UNESCO Publishing (2021).

